# Effects of adriamycin and candesartan on the collagen and elastin of the aorta in rats

**DOI:** 10.1186/2056-5909-1-2

**Published:** 2014-09-25

**Authors:** Jae-Sun Uhm, Woo-Baek Chung, Jung-Sook Yoon, Yong-Seog Oh, Ho-Joong Youn

**Affiliations:** Department of Cardiology, Severance Hospital, Yonsei University College of Medicine, Seoul, Korea; Department of Cardiology, Catholic University of Korea College of Medicine, Seoul, Korea; Clinical Research Center, Yeouido St Mary’s Hospital, Seoul, Korea

**Keywords:** Doxorubicin, Aorta, Candesartan, Collagen, Elastin

## Abstract

**Introduction:**

It has been reported that the chemotherapeutic agent, adriamycin, not only has an effect on the myocardium but also on the arteries. The aim of this study is to elucidate effects of adriamycin and an angiotensin receptor blocker, candesartan, on collagen and elastin of the aorta in rats.

**Methods:**

Twenty four male 8-week-old Wistar-Kyoto rats were divided into four groups: control (C) group, adriamycin-treated (AD) group, candesartan-treated (CA) group, and adriamycin- and candesartan-treated (AD + CA) group. Adriamycin of 2.5 mg/kg/wk was administered intraperitoneally one time per week for 6 weeks, and candesartan of 10 mg/kg/day was administered orally everyday for 6 weeks. After 6 weeks, the rats were sacrificed and the aortas were harvested. Hematoxylin-eosin staining, Verhoff’s elastic, and Goldner’s trichrome staining were performed for histopathologic analyses. Tunica media thickness, collagen, and elastic area fractions were measured quantitatively with a computerized digital image analyzer.

**Results:**

Tunica media thickness in the CA and AD + CA groups was significantly lesser than that in the C and AD groups, respectively. The AD and AD + CA groups had a tendency of lower elastin area fraction than the C and CA groups, respectively. Collagen area fraction in the AD + CA group was significantly lower than that in the AD group. There were no significant differences of collagen/elastin ratio between groups.

**Conclusions:**

These findings suggest that adriamycin has a tendency of decreasing the quantity of elastin fibers and candesartan cannot mitigate the effects of adriamycin on elastin fibers.

**Electronic supplementary material:**

The online version of this article (doi:10.1186/2056-5909-1-2) contains supplementary material, which is available to authorized users.

## Introduction

The anthracycline chemotherapeutic agent, adriamycin, is widely used and effective in many malignancies [1]. However, adriamycin has an important adverse effect, cardiotoxicity [2]. In practice, oncologists cannot often continue with the administration of adriamycin because of adriamycin-induced cardiomyopathy, though adriamycin is effective in inducing remission of cancer.

The aorta has the same embryologic origin as the heart, and it is anatomically and hemodynamically directly connected to the heart. The heart and the aorta often share the same pathophysiology of the diseases including hypertensive cardiovascular disease. Some researchers proposed that adriamycin has adverse effects not only on the heart but also on the arteries [3–5]. However, there have been not many studies on vascular pathology by adriamycin, and changes of aorta microstructure by adriamycin have not been known in detail. In addition, it has been reported that angiotensin-converting enzyme inhibitors or angiotensin receptor blockers can alleviate the fibrotic process [6]. This was a pilot study to investigate whether adriamycin can have an effect on collagen and elastin of the aorta in rats and whether candesartan can mitigate the effects of adriamycin.

## Methods

### Animals

**Figure 1:**
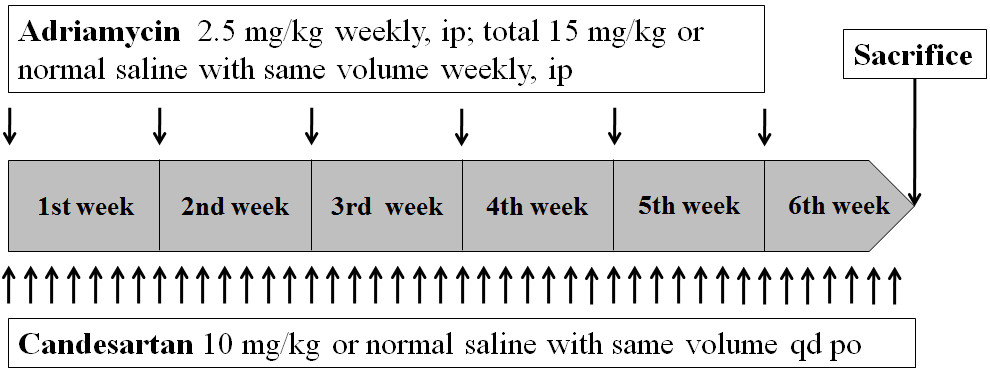
**Schedule of administration of adriamycin and candesartan.** ip, intraperitoneally; po, per os; qd, everyday.

The study protocol was approved by the institutional review board for Animal Research of Yeouido St. Mary’s Hospital (no: YEOUIDO-2012-13-01). Twenty four male 8-week-old Wistar-Kyoto rats were used. The rats were kept in cages under conditions of a room temperature of 20°C, a relative humidity of 60%, and a day/night cycle of 12 h. The rats had free access to food and water in cages. The rats were divided into four groups: control (C), adriamycin-treated (AD), candesartan-treated (CA), adriamycin- and candesartan-treated (AD + CA). The rats were weighed at the beginning of the experiment. In the AD and AD + CA groups, 2.5 mg/kg of adriamycin (Ildong Pharmaceuticals Co., Anseong, Korea) was injected intraperitoneally six times for 6 weeks (Figure 1) [7]. In the C and CA groups, normal saline of the same volume was injected by using the same method at the same time as the AD and AD + CA groups. In the CA and AD + CA groups, 10 mg/kg/day of candesartan (AstraZeneca, Zoetermeer, The Netherlands) was administered orally with a sonde (Figure 1). In the C and AD groups, normal saline of the same volume was administrated orally by using the same method at the same time as the CA and AD + CA groups. After 6 weeks, the rats were weighed and placed under anesthesia by zoletil (Virbac, Carros, France), the sternum was incised longitudinally, and the heart and the aorta were exposed. The rats were euthanized by injection of potassium chloride into both ventricles. After cardiac arrest, the aorta was extracted from the proximal ascending aorta to bifurcation of both iliac arteries.

### Staining and light microscopic examination

After the extracted aorta was irrigated with normal saline, the descending thoracic aorta was cut into small pieces. These pieces were fixed in 10% neutral-buffered formalin solution. After the dehydration process, the aortic tissue sample was embedded into a paraffin block and cut with a microtome. Hematoxylin-eosin staining, Verhoff’s elastin staining, and Goldner’s trichrome staining were performed for light microscopic examination. Elastin was stained black using the Verhoff’s elastin staining, and collagen was stained green using the Goldner’s trichrome staining. Photos of the aorta tissue were taken from six different areas per a rat at a magnification of × 400 with a light microscope (Zeiss, Jena, Germany). The photo images were quantitatively analyzed with Image-Pro Plus (MediaCybernetics, Silver Spring, MD, USA), a computerized digital image analyzer. Tunica media thickness was measured with a digital ruler and a microscopic scale in hematoxylin-eosin-stained section. The area in black was semi-automatically measured with Image-Pro Plus in Verhoff’s elastin-stained sections for measurement of the elastin area. The area in green was semi-automatically measured with Image-Pro Plus in Goldner’s trichrome-stained section for measurement of the collagen area. Elastin area fraction was calculated by dividing the elastin area by the total sampling area, and collagen area fraction was calculated by dividing the collagen area by the total sampling area. Collagen/elastin ratio was calculated by dividing the collagen area by the elastin area.

### Electron microscopic examination

The aortic tissue was fixed in 2.5% phosphate-buffered glutaraldehyde for 2 h and fixed in 1% osmium tetroxide and then dehydrated in serial alcohol. The aortic tissue was embedded in araldite and sectioned. After staining with toluidine blue, the aortic tissue sections were observed under microscope for precise location to cut for ultrathin sections. The endothelial and medial areas of the aorta were observed with an electron microscope (EM, Zeiss) at magnifications of × 8,000 and × 16,000, respectively.

### Statistical analysis

Because of nonnormal distribution of data, the results were expressed as median (interquartile range). Kruskal-Wallis test was used for comparison of baseline characteristics of continuous variables between the four groups. For *post hoc* analysis, we used Mann–Whitney *U* test with Dunnett comparisons between two groups: [8] the C group and the AD group, between the C group and the CA group, between the AD group and the AD + CA group, between the CA group and the AD + CA group, and between the C group and the AD + CA group. A *p* value and Dunnett-corrected *p* value (*p*_Dun_) <0.05 was considered statistically significant. The data were analyzed using the IBM SPSS ver. 20.0 (IBM Co., Armonk, NY, USA) and R Package ver. 2.0 (R Foundation for Statistical Computing, Vienna, Austria).

## Results

**Figure 2:**
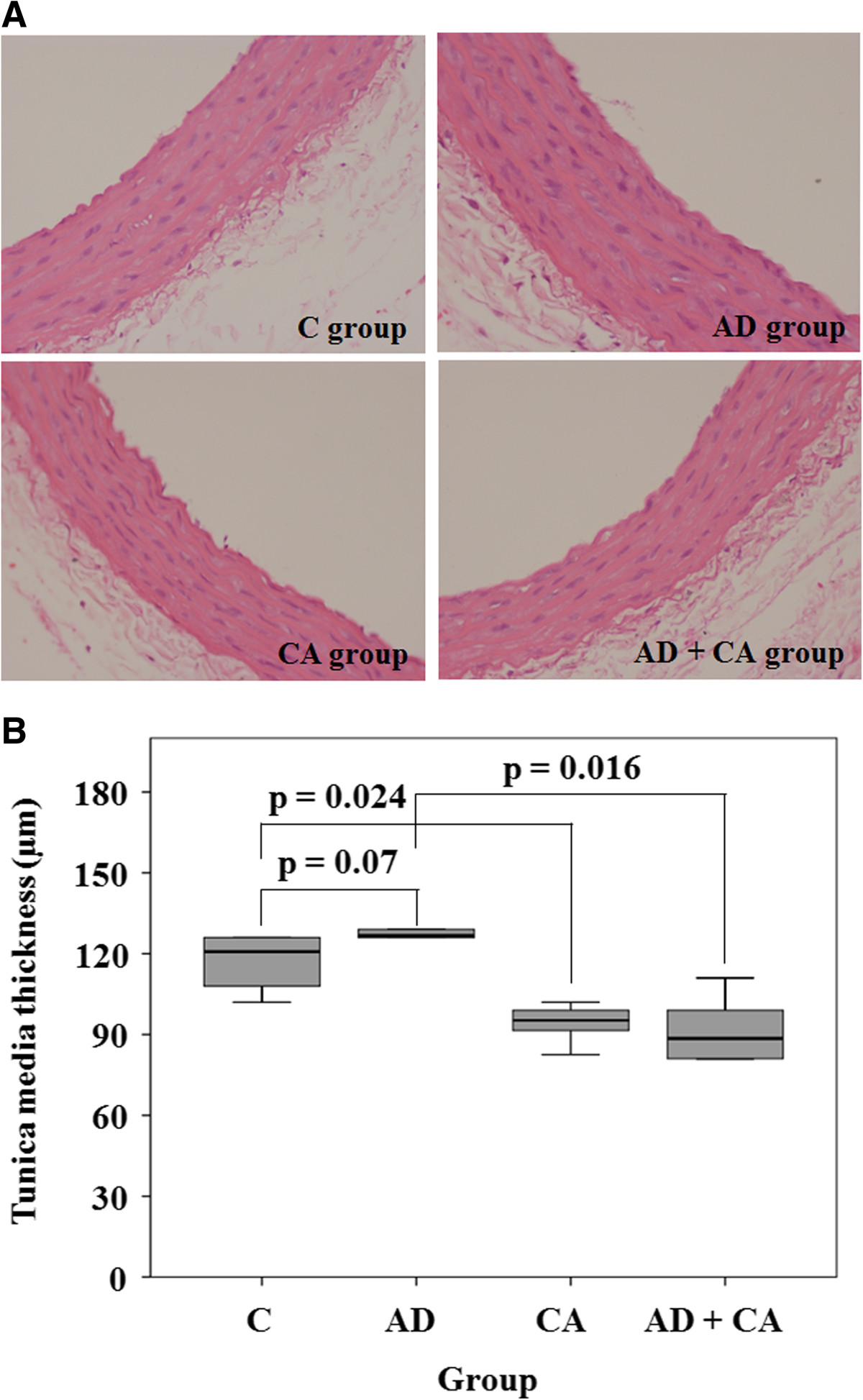
**Hematoxylin-eosin-stained section and tunica media thickness. (A)** Hematoxylin-eosin-stained section (×400) in C group, AD group, CA group, and AD + CA group. **(B)** Tunica media thickness within the aorta in each group. C, control; AD, adriamycin-treated; CA, candesartan-treated; AD + CA, adriamycin + candesartan-treated. *p* values were corrected by Dunnett method.

**Figure 3:**
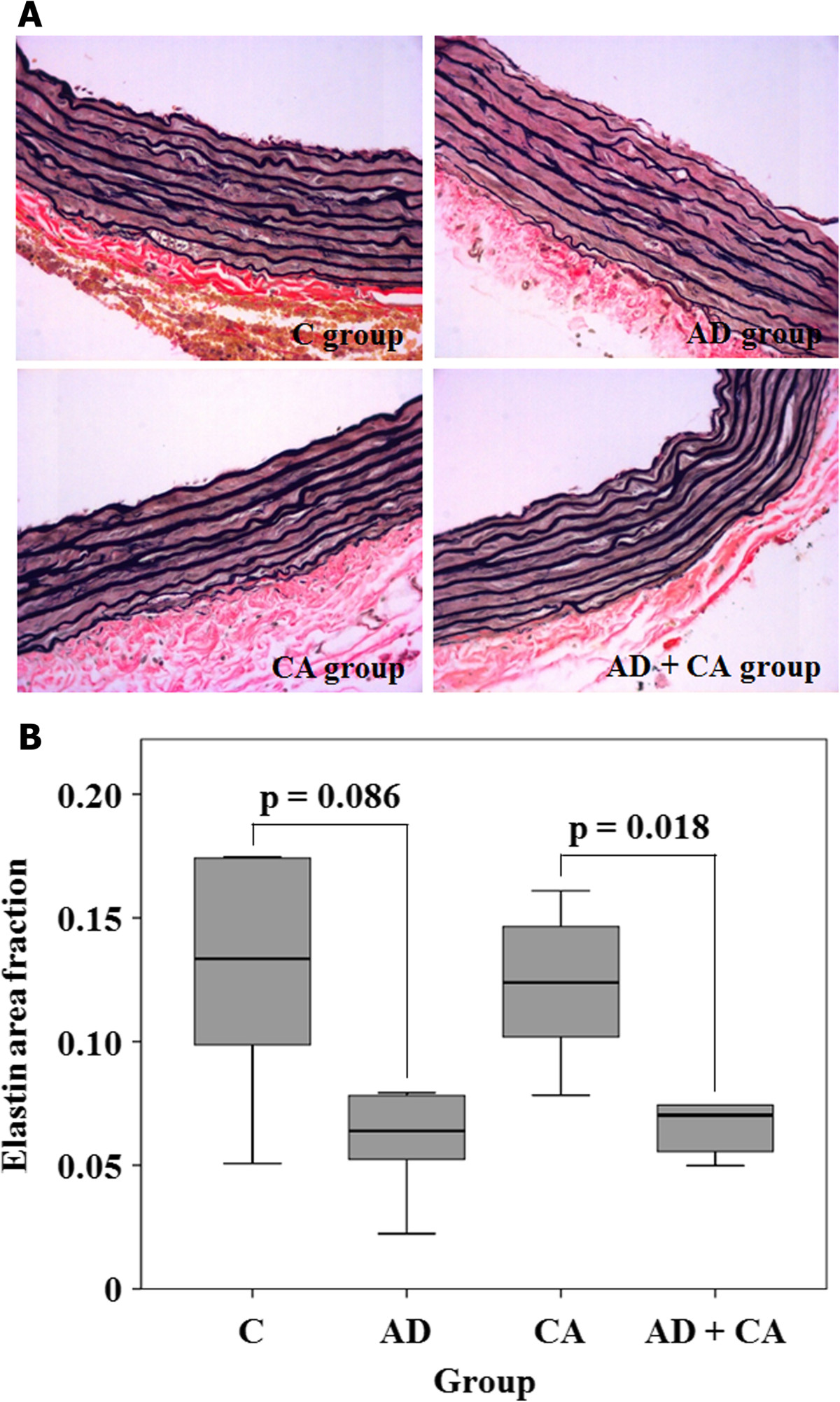
**Verhoff’s elastin stained section and elastin area fraction. (A)** Verhoff’s elastin-stained section (×400) in C group, AD group, CA group, and AD + CA group. **(B)** Elastin area fraction within the aorta in each group. C, control; AD, adriamycin-treated; CA, candesartan-treated; AD + CA, adriamycin + candesartan-treated. *p* values were corrected by Dunnett method.

**Figure 4:**
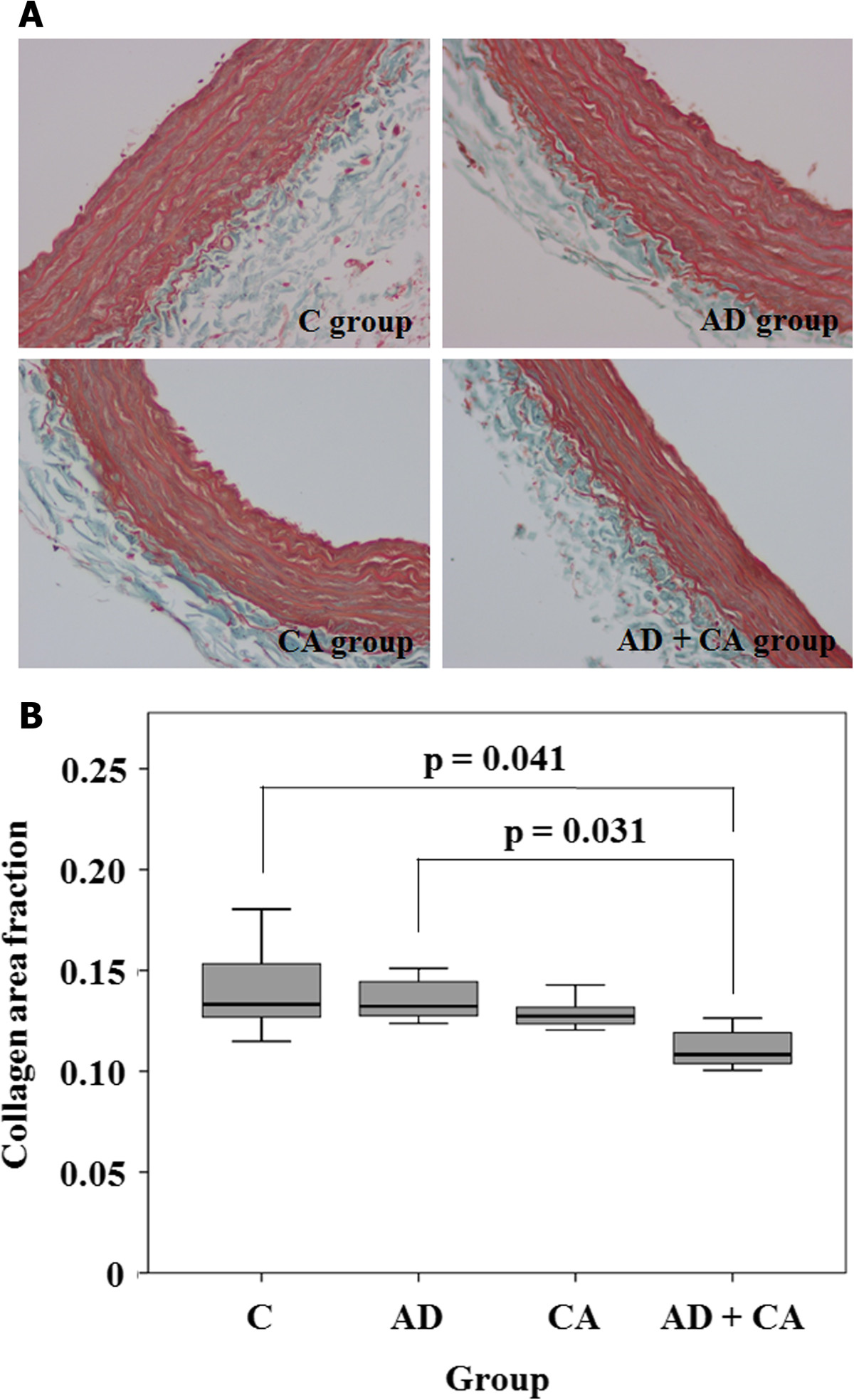
**Goldner’s trichrome stained section and collagen area fraction. (A)** Goldner’s trichrome-stained section (×400) in C group, AD group, CA group, and AD + CA group. **(B)** Collagen area fraction within the aorta in each group. C, control; AD, adriamycin-treated; CA, candesartan-treated; AD + CA, adriamycin + candesartan-treated. *p* values were corrected by Dunnett method.

**Figure 5:**
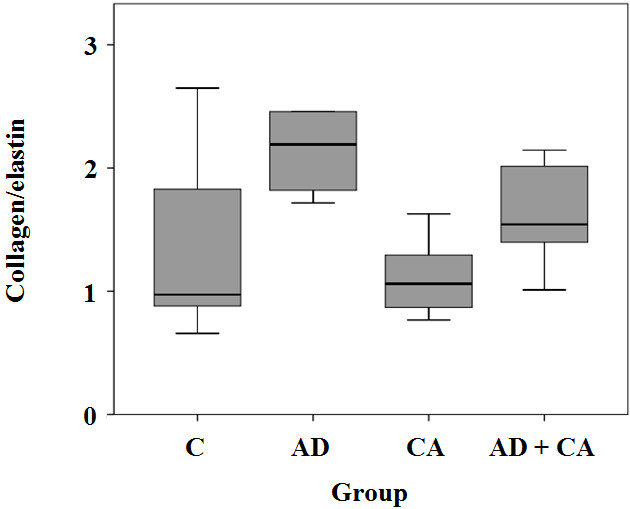
**Collagen/elastin within the aorta in each group.** C, control; AD, adriamycin-treated; CA, candesartan-treated; AD + CA, adriamycin + candesartan-treated.

**Figure 6:**
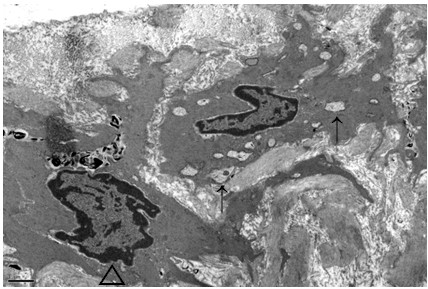
**Cytoplasmic vacuoles (arrow) and degenerative nucleus (arrow head) in the endothelial cells in the AD group.** Electron microscopy, ×8,000. AD, adriamycin-treated.

All of the 24 rats survived throughout the study period. There were no significant differences in body weights at the beginning of the study between the groups (*p* = 0.270) (Table 1). However, the rats in the AD group, the CA group, and the AD + CA group had a tendency to have lower weight gain compared with the rats in the C group, although there was no statistical significance (*p* = 0.223) (Table 1). There were no complications of intraperitoneal injection or oral administration. In hematoxylin-eosin-stained section, the tunica media thickness were 121.0 μm (range, 105.25 to 126.0 μm), 127.25 μm (range, 121.0 to 137.75 μm), 95.25 μm (range, 87.0 to 100.25 μm), and 89.0 μm (range, 80.75 to 104.75 μm) in the C group, the AD group, the CA group, and the CA + AD group, respectively. Tunica media thickness in the CA group was significantly lesser than that in the C group (*p*_Dun_ = 0.024). Tunica media thickness in the AD + CA group was significantly lesser than that in the AD group (*p*_Dun_ = 0.016) (Figure 2). However, there were no significant differences in tunica media thickness between the C group and the AD group and between the CA group and the AD + CA group. In Verhoff’s elastin-stained section, the elastin area fraction were 0.15 (range, 0.08 to 0.19), 0.07 (range, 0.04 to 0.09), 0.14 (range, 0.10 to 0.17), and 0.08 (range, 0.06 to 0.11) in the C group, the AD group, the CA group, and the CA + AD group, respectively. The AD group had a tendency of lower elastin area fraction than in the C group (*p*_Dun_ = 0.086). And the elastic area fraction in the AD + CA group was significantly lesser than that in the CA group (*p*_Dun_ = 0.018) (Figure 3). However, there were no significant differences in the elastin area fraction between the C group and the CA group and between the AD group and the AD + CA group. In Goldner’s trichrome-stained section, the collagen area fraction were 0.15 (range, 0.13 to 0.19), 0.15 (range, 0.14 to 0.16), 0.14 (range, 0.14 to 0.15), and 0.12 (range, 0.11 to 0.14) in the C group, the AD group, the CA group, and the AD + CA group, respectively. The collagen area fraction in AD + CA group was significantly lower than that in the C group and the AD group (*p*_Dun_ = 0.041 and 0.031, respectively) (Figure 4). Collagen/elastin ratio were 0.97 (range, 0.77 to 2.24), 2.19 (range, 1.77 to 4.09), 1.06 (range, 0.82 to 1.46), and 1.54 (range, 1.20 to 2.08) in the C group, the AD group, the CA group, and the CA + AD group, respectively. There were no significant differences in collagen/elastin ratio between the C group and the AD group (*p* = 0.093), between the C group and the CA group (*p* = 0.589), between the AD group and the AD + CA group (*p* = 0.240), and between the CA group and the AD + CA group (*p* = 0.180) (Figure 5). In electron microscopic examination of the endothelial cells, we observed intact endothelial layers of the aorta in nine rats (three rats of the C group, one rat of the AD group, three rats of the CA group, and two rats of the AD + CA group). There were no significant findings in the endothelial cells in the electron microscopic examination. In electron microscopic examination of the endothelial layers, there were cytoplasmic vacuoles and degeneration of nuclei in a rat in the AD group (Figure 6). No specific changes were detected in the other groups.

**Table 1:** **Changes of body weight before and after administration of drugs**

Group	BW1 (g)	BW2 (g)	ΔBW/BW1 × 100 (%)
C	180.5 (164–192.5)	289.5 (265.5–296.5)	60 (54–62)
AD	186.5 (186–194.5)	262 (247–266)	40 (27–43)
CA	170.5 (167–190.5)	235.5 (239.5–287)	38 (26–71)
AD + CA	172.5 (158–174.5)	224 (210–244.5)	40 (22–42)

*BW1* body weight before administration of drugs, *BW2* body weight after administration of drugs, *ΔBW* BW2 - BW1, *C* control, *AD* adriamycin-treated, *CA* candesartan-treated.

## Discussion

This pilot study demonstrated that the adriamycin-treated groups had a tendency of lower elastin area fraction than the adriamycin-untreated groups. And candesartan decreased the thickness of tunica media in the aorta in adriamycin-treated and adriamycin-untreated rats and the quantity of collagen fibers in the aorta of adriamycin-treated rats. According to the results of the present study, adriamycin had a tendency of reduced quantity of elastin fibers in the aorta. As a result, elastance of the aorta could decrease and aortic stiffness could increase [9]. Decreased elastance and increased stiffness of the aorta mean pathologic processes and structural remodeling of the aorta. It could aggravate hypertension and cause target organ damage including left ventricular hypertrophy [10, 11]. Decrement of aortic elastance could result in aggravation of adriamycin-induced cardiotoxicity by myocardial fibrosis and structural remodeling. In addition, candesartan decreased the tunica media thickness and the quantity of collagen fibers in the aorta in the present study. It was because, generally, angiotensin receptor blockers have the effects of mitigating fibrosis by decreasing thickness of tunica media and collagenogenesis. Reduction of the quantity of collagen fibers means mitigation of fibrosis and reverse of structural remodeling of the aorta. Decrease of tunica media thickness could be relative to reverse of structural remodeling of the aorta. However, candesartan could not show the mitigation of decrement of elastin fibers by adriamycin.

Because fibrosis plays an important role in cardiomyopathy and vasculopathy, drugs that have an effect on the fibrotic process are essential for the treatment of cardiomyopathy and vasculopathy. Angiotensin converting enzyme inhibitors or angiotensin receptor blockers can regulate the renin-angiotensin-aldosterone system and ameliorate the fibrotic process [6]. In addition, it was reported that spironolactone, an aldosterone receptor blocker, and torasemide, a loop diuretic, can reduce fibrosis [12, 13].

Because the aorta is directly connected to the heart, the heart and aorta often share the same pathophysiologic processes. It has been reported that adriamycin induces aortic toxicity [14]. However, the mechanisms of adriamycin-induced aortopathy are not known in detail. The suggested mechanisms of adriamycin-induced vasculopathy were endothelial dysfunction [15–18], suppression of endothelin-1 in endothelial cells [19], degeneration of endothelial structures [14], apoptosis of the endothelial cells [20], enhancement of procoagulant activity [21], smooth muscle dysfunction, and dysregulation of its calcium contents [10, 22]. In addition, adriamycin could induce not only cardiovascular toxicity but also nephrotoxicity by alteration of glomerular endothelial cells [16, 23].

Usually, injection of adriamycin induces retardation of weight gain [7]. In this study, adriamycin- or candesartan-treated rats reduced weight gain compared with rats in control group, although there was no statistical significance. In adriamycin-treated rats, it could be because adriamycin has systemic toxicity. It has reported that incidence of gastrointestinal adverse effects of candesartan was 1% in human study [24]. In adriamycin-treated rats, reduced weight gain might be due to gastrointestinal adverse effects of candesartan.

We have shown in the previous report that collagen in the myocardium significantly increased in adriamycin-induced cardiomyopathy rat model [7]. However, collagen area fraction between the C group and the AD group was not significantly different in the present study. It could be because the mechanisms of aortopathy could be different from those of cardiomyopathy and because the numbers of rats and the duration from the drug administration to sacrifice could be short.

In this study, ultramicroscopic structural changes were not obvious. It might be because the adriamycin-induced microscopic changes in the aorta were slower than those in cardiomyopathy due to the extracellular matrix of the aorta. It might be because the rats in the present study were too young to present the microscopic pathologic processes including atherosclerotic changes.

We had some limitations of the present study. We could not confirm the development of cardiomyopathy because we did not perform echocardiography in the rats. We could not show microscopic and ultramicroscopic changes of endothelial cells by adriamycin and candesartan because the endothelial layers were damaged in the processes of fixation and staining.

## Conclusions

Adriamycin had a tendency of decreasing the quantity of elastin fibers, and candesartan cannot mitigate the effects of adriamycin on elastin fibers. In oncology clinic, physicians should be cautious about not only adriamycin-induced cardiotoxicity but also adriamycin-induced vascular changes. Further studies are necessary to evaluate the effects of angiotensin-converting enzyme inhibitors or angiotensin receptor blockers on the adriamycin-induced vascular changes.

## Authors’ original submitted files for images

Below are the links to the authors’ original submitted files for images. Authors’ original file for figure 1 Authors’ original file for figure 2 Authors’ original file for figure 3 Authors’ original file for figure 4 Authors’ original file for figure 5 Authors’ original file for figure 6

## Abbreviations

AD: Adriamycin
C: Control
CA: Candesartan
*P*_Dun_: Dunnett-corrected *p* value.
