# Erratum to: Effects of adriamycin and candesartan on the collagen and elastin of the aorta in rats

**DOI:** 10.1186/s40885-015-0023-8

**Published:** 2015-07-01

**Authors:** Jae-Sun Uhm, Woo-Baek Chung, Jung-Sook Yoon, Yong-Seog Oh, Ho-Joong Youn

**Affiliations:** 1 Department of Cardiology, Severance Hospital, Yonsei University College of Medicine, Seoul, Korea; 2 Department of Cardiology, Catholic University of Korea College of Medicine, Seoul, Korea; 3 Clinical Research Center, Yeouido St Mary’s Hospital, Seoul, Korea; 4 Department of Cardiology, Cardiovascular Center, Seoul St. Mary’s Hospital, 222 Banpo-daero, Seocho-gu, Seoul 137-701 Korea

After publication of the article [1] it was discovered that a citation error had occurred, resulting in different citation numbers in the PDF and HTML files. The correct citation of 20:8 has now been updated in all versions of the manuscript. We apologise for any inconvenience caused by this error.
