# Supplementary figures and images for: Effects of adriamycin and candesartan on the collagen and elastin of the aorta in rats
Source: Clin Hypertens. 2014 Sep 25;20:8. doi: 10.1186/2056-5909-1-2 (PMC4763430; doi:10.1186/2056-5909-1-2)

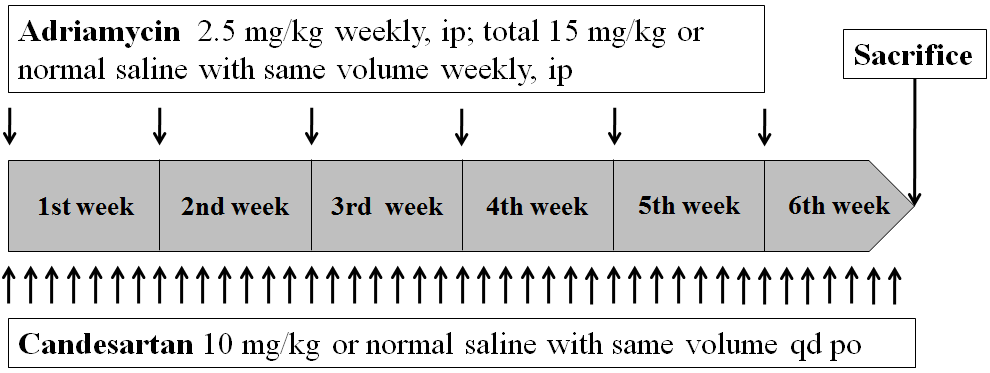

Supplement: Supplementary file 1 — Authors’ original file for figure 1 [file 40885_2014_2_MOESM1_ESM.png]

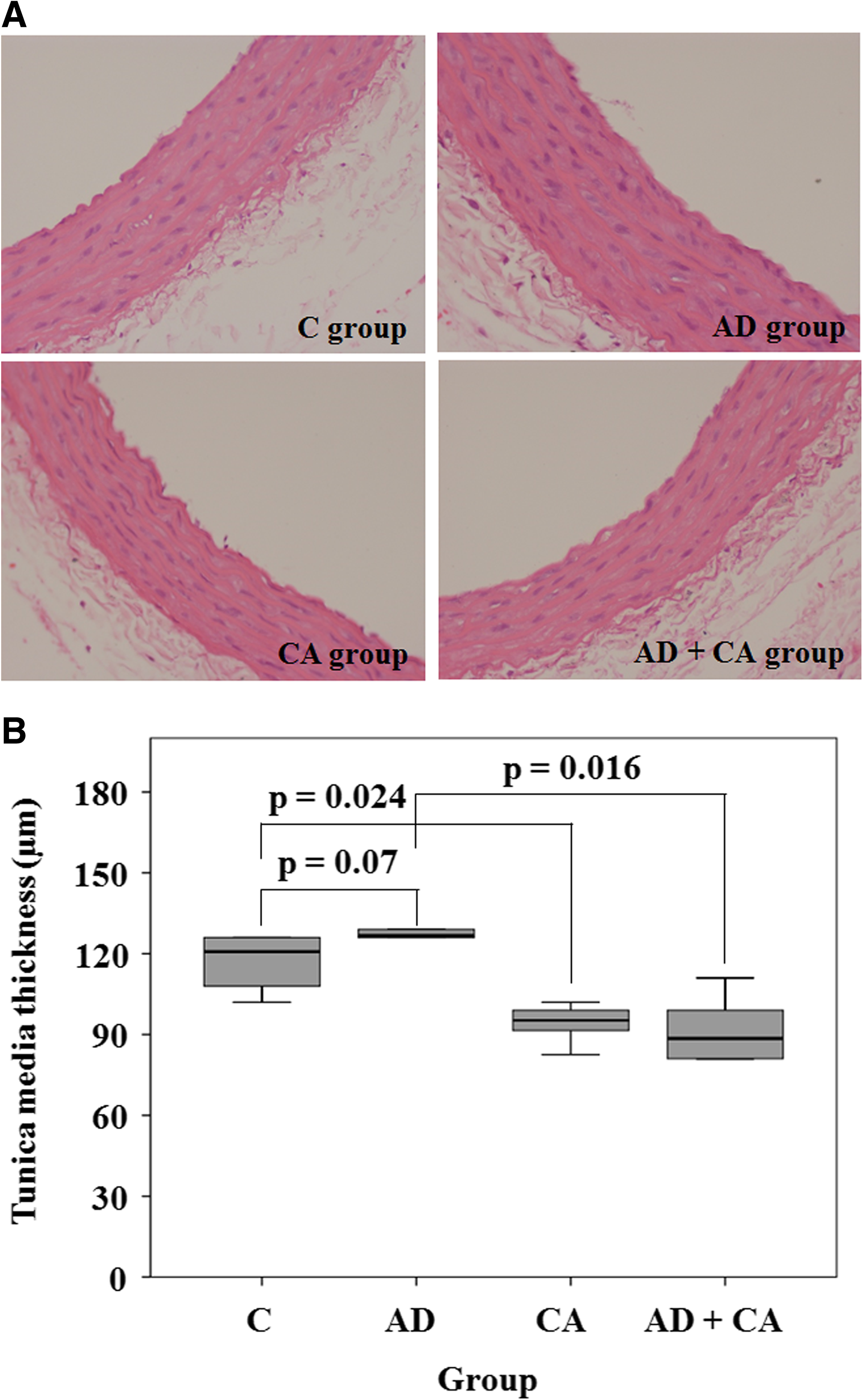

Supplement: Supplementary file 2 — Authors’ original file for figure 2 [file 40885_2014_2_MOESM2_ESM.tiff]

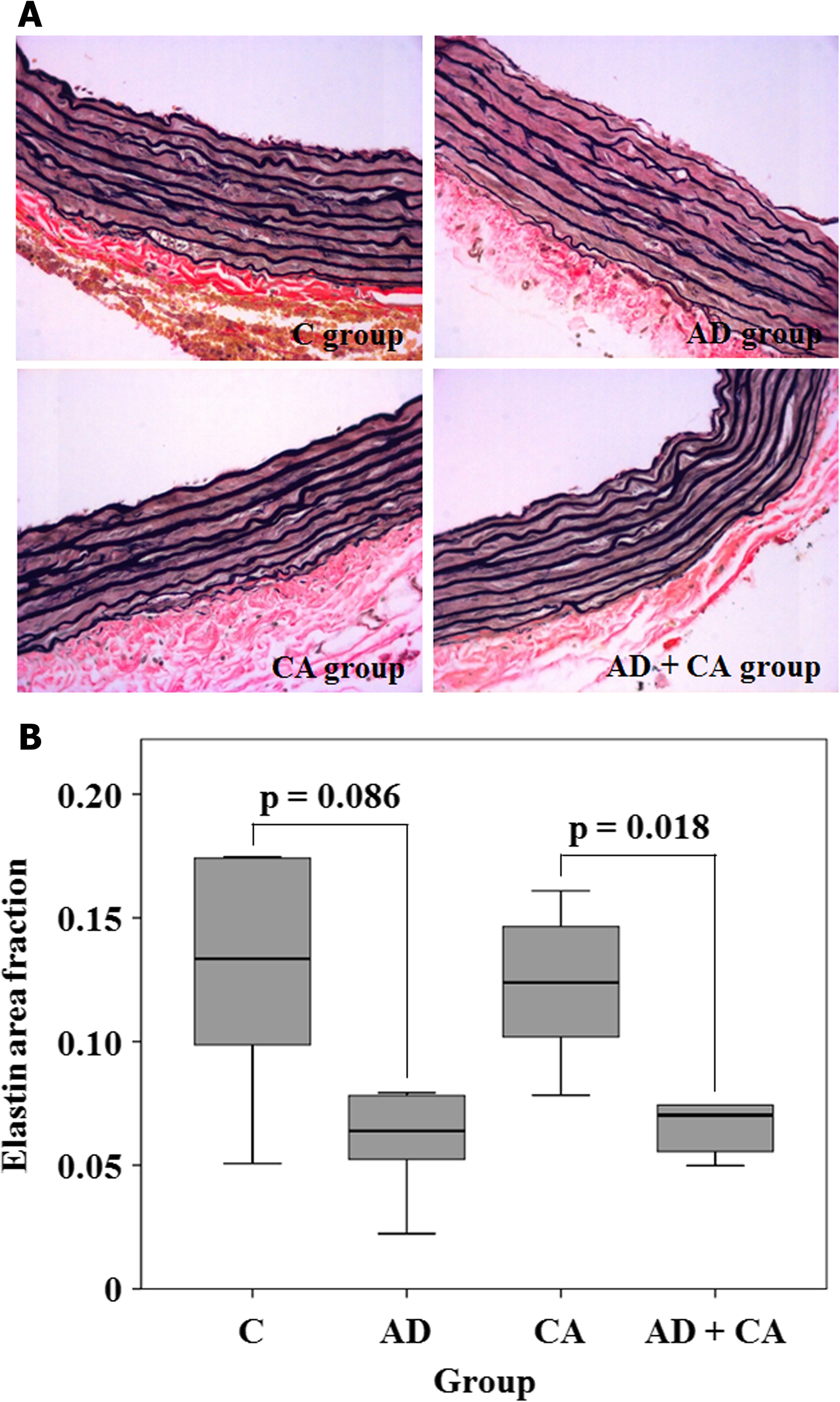

Supplement: Supplementary file 3 — Authors’ original file for figure 3 [file 40885_2014_2_MOESM3_ESM.tiff]

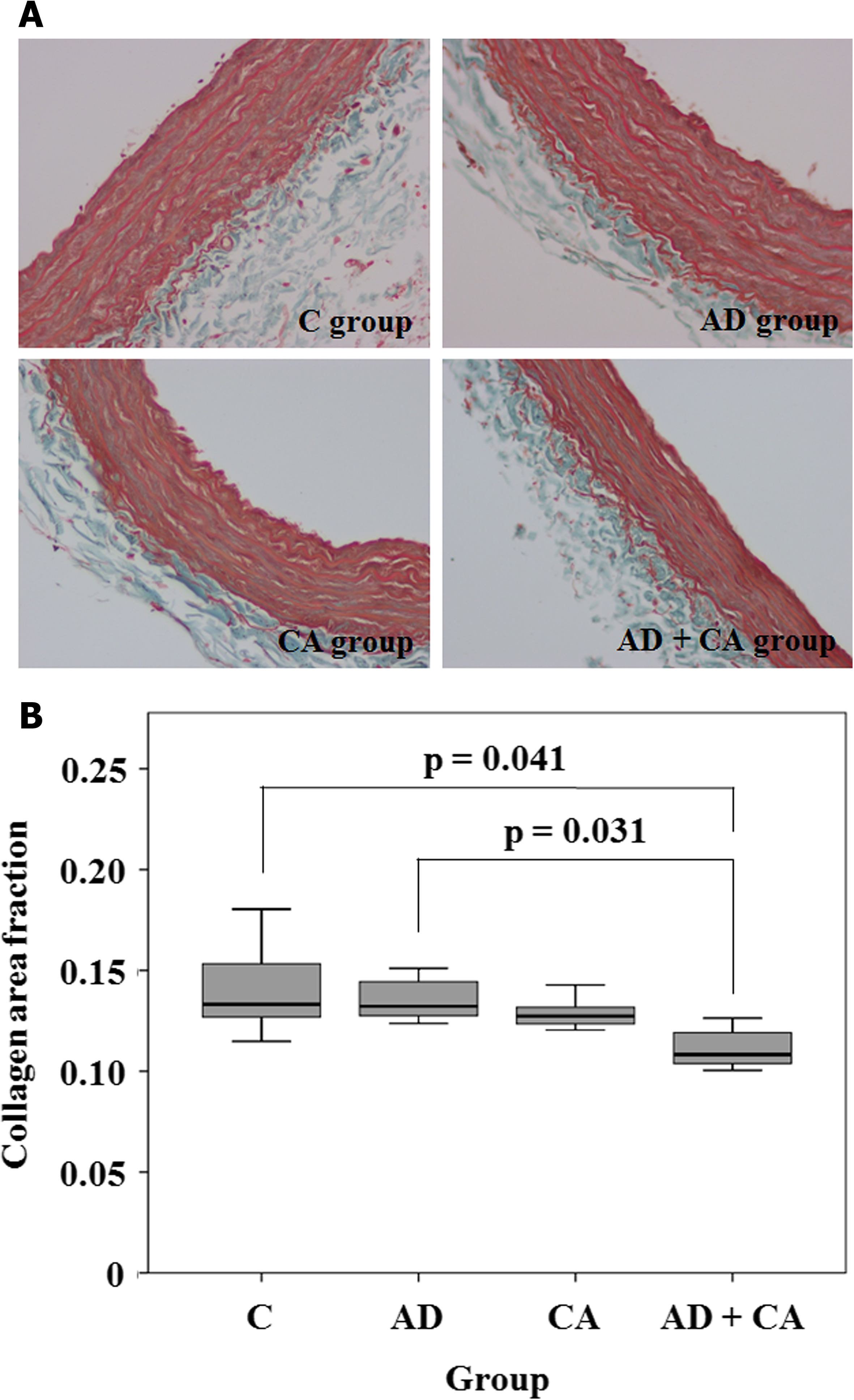

Supplement: Supplementary file 4 — Authors’ original file for figure 4 [file 40885_2014_2_MOESM4_ESM.tiff]

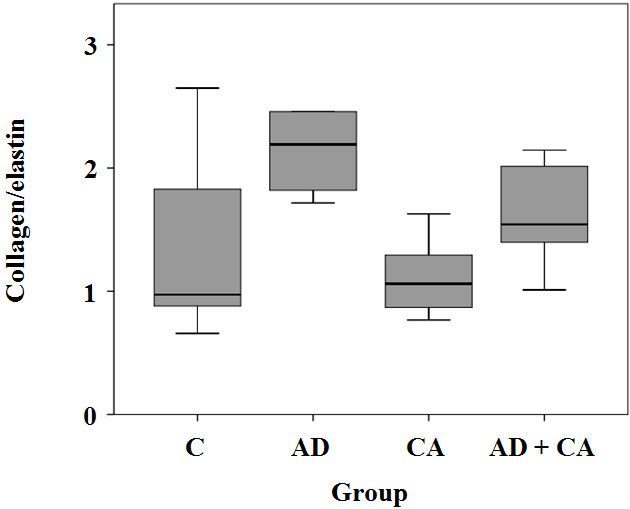

Supplement: Supplementary file 5 — Authors’ original file for figure 5 [file 40885_2014_2_MOESM5_ESM.png]

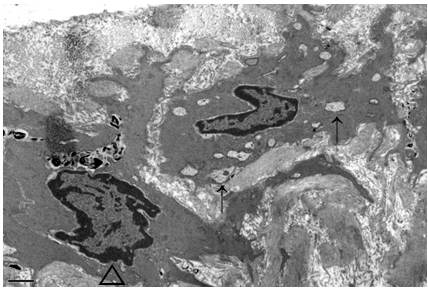

Supplement: Supplementary file 6 — Authors’ original file for figure 6 [file 40885_2014_2_MOESM6_ESM.png]
